# Comparative Analysis of Indoor and Outdoor Particulate Matter Concentrations and Air Quality in Ogbomoso, Nigeria

**DOI:** 10.5696/2156-9614-10.28.201205

**Published:** 2020-11-19

**Authors:** Musibau O. Jelili, Adeniyi S. Gbadegesin, Abimbola T. Alabi

**Affiliations:** Department of Urban and Regional Planning, Faculty of Environmental Sciences, Ladoke Akintola University of Technology, Ogbomoso, Oyo State, Nigeria

**Keywords:** air quality, particulate matter, residential zones

## Abstract

**Background.:**

Airborne particulates are an issue in many urban regions around the world and their detrimental impact on human health has increasingly become a public health concern.

**Objectives.:**

The aim of the present study was to examine particle pollution in an urban settlement in Nigeria. This study examines the extent, spatial variation, and sources of indoor and outdoor particulate matter (PM) concentrations in Ogbomoso, Nigeria.

**Methods.:**

The survey research method was adopted. Sampling included 385 buildings across selected precincts and different residential zones in the town of Ogbomoso. Particulate matter analytes (PM_1_, PM_2.5_ and PM_10_) within/around each building were measured with a particle counter and details on domestic utilities/practices were obtained with a questionnaire. Analysis of variance was used to determine inter-zonal variations in PM levels and simple linear regression was used to analyze the relationship between indoor and outdoor air quality.

**Results.:**

Indoor and outdoor respirable particle (PM_2.5_) concentrations were lower than the World Health Organization (WHO) Interim Target limit of 75 μg/m^3^, while concentrations of inhalable particles (PM_10_) were higher than the set limit of 150 μg/m^3^ for daily averages. Coarse particles dominated, with an accumulative PM_2.5_/PM_10_ ratio of 0.24. The inter-zonal analysis of PM concentrations revealed that indoor and outdoor PM levels varied significantly by residential zone (p = 0.0005; p = 0.01, respectively). Regression analysis showed a significant but weak relationship between indoor and outdoor PM levels (r = +0.221), while the coefficient of determination (R^2^ = 0.049) showed that only about 5% of the variation in indoor air quality was associated with outdoor air quality. Particle pollution inducers were identified in the residents' waste disposal methods and adopted fuels/energy sources, with firewood and charcoal linked with increased concentrations of particulate matter.

**Conclusions.:**

Air quality was relatively poor in the study area given observed particulate matter concentrations. Cleaner fuels, effective waste management systems and improved roads are needed to foster better air quality in the study area.

**Competing Interests.:**

The authors declare no competing financial interests

## Introduction

Air pollution has been a serious environmental problem for many years and is becoming a greater issue globally. Particulate matter in ambient air presents a risk to human health, particularly after prolonged exposure. Air quality problems are experienced in both developed and developing countries and epidemiological studies have demonstrated correlational and causal relationships with various ailments. The International Agency for Research on Cancer (IARC) revealed that worldwide 223,000 lung cancer deaths in 2010 resulted from air pollution.^1^ Air pollution is considered the world's largest single environmental health risk, responsible for one in eight total global deaths.^2^

Both indoor and outdoor air pollution present a major challenge, although studies often show mortality from indoor air pollution to be twice that of outdoor air pollution.^3–5^ This problem is even greater in developing countries. According to the World Health Organization (WHO), more than two-thirds of the global estimates of mortality from indoor air pollution occur in Southeast Asia and sub-Saharan Africa.^6^ In Nigeria, the use of biomass cooking fuels, unclean energy sources, unsanitary waste disposal techniques and other activities which release gaseous and particulate pollutants into the indoor and outdoor environments are common. This highlights the need for the examination of air quality in urbanizing settlements in Nigeria.

Within residential environments, ambient particulate matter (PM) is a useful measure of air quality. Particulate matter includes a wide variety of substances including dust, pollen, dander, airborne smoke, and soot as well as liquid droplets from fuel combustion.^7^ According to the World Bank, the amount of suspended PM, usually measured in micrograms per cubic meters of air, is one of the most important indicators of air quality.^8^ There are often differences in the constituents of indoor and outdoor air pollution according to pollution sources, yet airborne particulates are notably common^9^, making PM a good indicator for examining differences between indoor and outdoor air quality.

There is a growing need for empirical investigations into indoor and outdoor air quality with regard to airborne particulates. Few such studies have been conducted in developing countries. Air quality studies in Nigeria often address either indoor air quality in rural areas or outdoor air quality in industrialized regions, addressing primarily solid fuel use in the former and vehicular/industrial emissions in the later. There have been few studies set in residential urbanizing settlements and still fewer exploring differences between indoor and outdoor air quality. The present study examines air quality in Ogbomoso, Nigeria and provides insight into the extent, spatial variation and interconnection between indoor and outdoor air quality.

### Particulate matter

Particulate matter is used to describe condensed phase (solid or liquid) particles suspended in the atmosphere and it is categorized by size, specifically by aerodynamic diameter in microns (millionths of a meter).^10^ The potential for causing health problems is directly linked to the size of the particles; small particles are generally thought to be more damaging to health.^3^ Three PM analytes are often considered in air quality studies viz: PM_10_ (coarse particles less than 10 μm), PM_2.5_ (fine particles less than 2.5 μm) and PM_1_(ultrafine particles between 0.1 and 1 μm), and their health implications are well documented. The fine particles of PM_2.5_ (respirable particles) pass through the nose and are deposited in the windpipe or lungs, where as PM_10_ (inhalable particles) are deposited on the hairs in the nose or at the bends of the nasal passages; both are causal factors for respiratory and cardiovascular morbidity.^11^ However, PM_2.5_ is a stronger risk factor for mortality than PM_10_, while PM_1_ which is smaller in size, is considered more harmful than both as it is likely to reach deeper into the respiratory system carrying with it more toxins from anthropogenic emissions.^12,13^

**Abbreviations**
*CPMag*: Cumulative particulate matter aggregate
*I/O*: Indoor/outdoor
*PM*: Particulate matter
*WHO*: World Health Organization

Long term exposure to particulate matter has been linked with acute respiratory morbidity in the form of pneumonia and asthma.^5,8,14^ Diesel exhaust—a major contributor to PM pollution—has been linked to acute vascular dysfunction and increased thrombus formation.^15^ Moreover, air pollution from fine particulates (PM_2.5_) is estimated to be responsible for about 3% of adult cardiopulmonary disease mortality; about 5% of trachea, bronchus, and lung cancer mortality; and about 1% of mortality in children from acute respiratory infection in urban areas worldwide. According to Cohen *et al.*, this amounts to about 0.8 million (1.2%) premature deaths and 6.4 million (0.5%) lost life years.^4^

Recently, studies in Nigeria have explored the extent of PM pollution. Particulate loads have been found to range between 40 μg/m^3^ and 98 μg/m^3^ in sixteen Niger Delta communities, exceeding the World Bank standards for annual mean.^16^ Total suspended particles have similarly been found to exceed existing (Nigerian) Federal Environmental Protection Agency's standards in the Niger Delta region.^17^ A study in Lagos using an air quality index derived from computations with set standards revealed very poor air quality from suspended PM across selected sampling locations and zones within the state.^18^ Furthermore, a cross sectional study across six Nigerian megacities (Aba, Abuja, Lagos, Kano, Maiduguri and Port Harcourt) showed that daily PM_10_ mass loads exceeded the WHO set limits, with the exception of Abuja, while Aba had the highest PM_10_ concentration levels.^19^ Most of these studies have, however, focused on metropolitan areas, especially in the Niger Delta region of Nigeria.

## Methods

**Figure 1:**
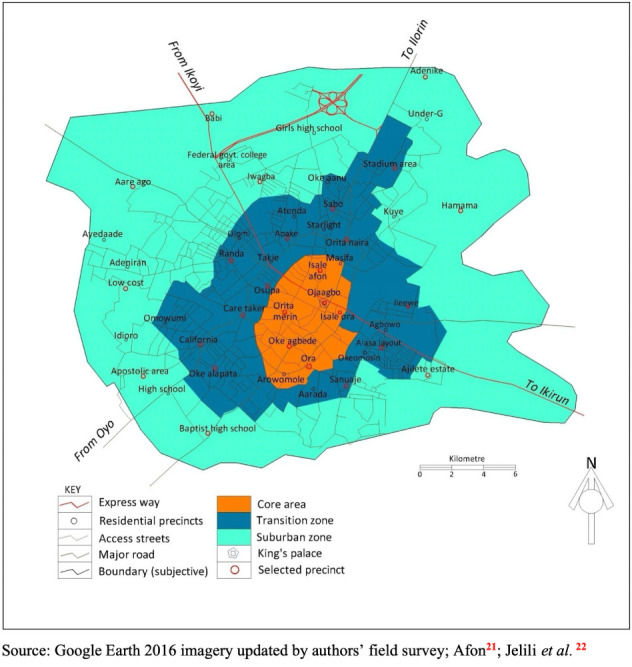
Map of the study area showing sampled precincts and residential zones

The present study took place in Ogbomoso, an urbanizing town in southwestern Nigeria, and covered Ogbomoso North and Ogbomoso South Local Government Areas in 2016. With an estimated population of 354,617,^20^ these two local government areas in the Ogbomoso Region constitute Ogbomoso Township. Ogbomoso Township can be delineated into three major residential zones (core, transition and suburban) largely stemming from the pre-colonial, colonial and post-independence phases of the development in the country, respectively, and form the ecological zones of a traditional African city.^21^ The core residential zone, which is the innermost and oldest part of the town, consists of the king's palace, some traditional landmarks, the king's market and buildings connected mostly by footpaths and narrow access roads. People indigenous to the town dominate the area. The houses are built in compounds, usually circular in shape, with a large central area where families meet or play, although this quintessential structure is being slowly being eroded by some newly built buildings. Small-scale retail and indigenous business outfits are the most common activities in this zone. Surrounding the core area is the transition (intermediate) zone. This zone houses the growing numbers of middle-income residents, primarily employed in the formal sector. The transition zone is the bridge between the core and suburban zones. It has a greater proportion of tenement houses, a higher population density than the other two zones and is dominated by mixed wholesale commercial and tertiary activities. The last zone, the suburban zone, is also called the commuter zone. This zone lies along lines of rapid transit beyond the city limits and is essentially an area of high-income developments, often landscaped and well laid-out and inhabited mostly by high income earners. The above-mentioned ecological zones were used to organize this study *(Figure 1).*

Following the design of a previous study, “Intra-urban pattern and determinants of indoor air quality in Ogbomoso, Nigeria”^22^, the survey covered 54 residential precincts scattered across the township, half of which were selected for sampling but evenly spread to prevent clusters and ensure representativeness *(Figure 1).*^22^ Twenty-seven (27) precincts were selected with 6, 12 and 9 in the core, transition and suburban zones, respectively (a ratio of 2:4:3), based on their respective human and housing population sizes. Given the need to collect indoor and outdoor PM data simultaneously, residential buildings were used as a basis for data collection. Buildings in the selected precinct were counted using Google Earth. A total of 10% of the buildings were selected for sampling via random systematic sampling, using an interval of eight alternately along both sides of the road. Ethical approval was granted from the Postgraduate Committee of the Department of Urban and Regional Planning, Ladoke Akintola University of Technology at a pre-data postgraduate seminar.

A total of 385 buildings (73,187 and 125) from the core, transition and suburban zones, respectively, were sampled and data collected over a period of four weeks during the rainy season. In each building, a questionnaire was administered to an adult resident and the GT-531 mass particle counter (Met One Instruments, Inc.) was used for PM data collection. Consent was obtained for the survey in buildings as residents were informed of the nature of the study and the safety of the particle counter prior to requests to take air samples and administering the questionnaire *(Supplemental Material).* The questionnaire was designed to elicit information on housing characteristics, domestic utilities and other practices. Data collection included building type, ventilation, cooking practices, waste disposal and household energy use. The PM_1_, PM_2.5_ and PM_10_ levels in the living room and immediate surroundings were obtained with a particle counter. Recorded PM concentrations were then computed, and aggregate scores compared with WHO standards. Base-10 log transformation was used for normalizing obtained PM data.^23^ Analysis of variance and correlation were carried out to analyze the inter-zonal variations of indoor and outdoor air quality, as well as the relationship between both.^23^

## Results

The indoor and outdoor mean concentrations for PM_1_, PM_2.5_ and PM_10_were computed and presented per precinct in Table 1. For ultrafine particles, the highest mean indoor and outdoor readings were 32.3 μg/m^3^ and 35.5 μg/m^3^, respectively. For fine particles, the highest mean indoor reading was 51.4 μg/m^3^ and highest outdoor reading was 60.5 μg/m^3^. For coarse particles, the highest mean indoor and outdoor readings were 230.8 μg/m^3^ and 382.7 μg/m^3^, respectively. The cumulative mean indoor and outdoor PM_1_, PM_2.5_ and PM_10_ for the study area were 23.6 μg/m^3^ and 27.7 μg/m^3^; 41.6 μg/m^3^ and 46.3 μg/m^3^; and 175.5 μg/m^3^ and 188.8 μg/m^3^, respectively. With regard to PM_2.5_/PM_10_ ratio, the cumulative mean for the study area was 0.24 *(Table 2)* while the indoor/outdoor (I/O) PM ratios for the ultrafine, respirable and inhalable particles were 0.84, 0.90 and 0.92 *(Table 3)*.

**Table 1:** Indoor and Outdoor PM Concentrations

**Zone**	**No.**	**Selected Precincts**	**Sampled buildings**	**Outdoor mean (μg/m^3^)**			**Indoor mean (μg/m^3^)**		

				**PM_1_**	**PM_2.5_**	**PM_10_**	**PM_1_**	**PM_2.5_**	**PM_10_**
Core area	1	Oja Igbo	13	30.9	44.9	172.8	26.6	45.3	168.1
	2	Isale Ora	13	35.1	58.4	328.5	24.3	44.8	178.0
	3	IsaleAfon	15	25.7	40.9	173.5	25.8	41.7	177.3
	4	Orita Merin	9	24.9	41.0	172.1	26.3	45.4	200.8
	5	OkeAgbede	15	26.4	43.8	182.2	24.1	41.8	154.5
	6	Ora	8	31.1	46.1	189.9	24.2	47.6	186.3
Transition	7	Ileewe	22	33.6	38.4	187.9	24.2	41.3	154.9
	8	Alasa layout	12	31.2	50.7	253.3	32.3	51.4	230.8
	9	Sanuaje	20	28.4	54.0	214.5	25.6	48.8	189.4
	10	OkeAlapata	10	22.3	42.3	154.7	22.9	40.3	159.6
	11	California	16	23.6	36.9	162.9	22.2	36.2	172.3
	12	Care Taker	15	23.0	36.2	140.5	26.6	41.7	184.9
	13	Osupa	11	30.7	56.8	267.7	25.1	49.6	218.9
	14	Randa	7	28.8	53.9	278.6	22.6	46.3	220.8
	15	Apake	13	30.9	54.2	382.7	22.9	40.9	189.2
	16	Sabo	7	31.8	46.0	208.5	23.2	36.1	194.7
	17	Stadium	48	26.2	42.6	150.2	25.1	40.3	195.8
	18	Orita Naira	6	35.5	60.5	314.4	22.9	48.9	221.5
Suburban	19	Ajilete Estate	7	17.8	26.5	96.8	17.9	28.7	168.7
	20	Iwagba	13	27.6	48.8	164.3	20.0	37.6	170.5
	21	Baptist High Scl.	14	27.7	45.8	168.0	20.6	36.9	148.8
	22	Babi	11	21.9	35.3	136.0	19.8	32.3	144.8
	23	Hamama	21	29.3	52.0	193.8	22.3	45.1	157.3
	24	Adenike	30	26.5	42.1	149.8	20.9	37.5	154.7
	25	Aare Ago	8	31.3	55.9	179.4	21.1	48.1	145.8
	26	Low Cost	7	23.9	36.4	98.2	18.8	30.3	142.7
	27	Apostolic	14	23.7	42.1	162.8	23.7	40.4	145.7
	Cumulative mean		385	27.7	46.3	188.8	23.6	41.6	175.5

Sampling time range per building: 45 minutes-1 hour.

**Table 2:** PM_2.5_/PM_10_ Ratios in Ogbomoso

**Mean (μg/m^3^)**	**Core**			**Transition**			**Suburban**		

	**PM_2.5_**	**PM_10_**	**PM_2.5/10_**	**PM_2.5_**	**PM_10_**	**PM_2.5/10_**	**PM_2.5_**	**PM_10_**	**PM_2.5/10_**
Indoor	43.8	174.3	0.25	42.8	190.6	0.22	38.4	153.8	0.25
Outdoor	46.9	212.7	0.22	47.6	2008.	0.24	43.9	157.0	0.28
Total	90.7	387.0	0.23	90.4	391.4	0.23	82.3	310.8	0.26
Cumulative mean*	0.24								

^*^PM_2.5_/PM_10_ cumulative mean ratio for the study area

**Table 3:** Indoor/Outdoor PM Ratios in Ogbomoso

**Residential zone**	**PM_1_**			**PM_2.5_**			**PM_10_**		

	**Indoor (μg/m^3^)**	**Outdoor (μg/m^3^)**	**I/O**	**Indoor (μg/m^3^)**	**Outdoor (μg/m^3^)**	**I/O**	**Indoor (μg/m^3^)**	**Outdoor (μg/m^3^)**	**I/O**
Core area	24.8	29.5	0.84	43.8	46.9	0.93	174.3	212.7	0.82
Transition	25.0	27.9	0.90	42.8	47.6	0.90	190.6	200.8	0.95
Suburban	20.9	26.2	0.79	38.4	43.9	0.87	153.8	157.0	0.98
Mean			0.84			0.90			0.92

### Observed PM pollution inducers

Table 4 presents the housing or building characteristics in the study area, which were mainly rooming types (57.7%). Flats constituted 27.5% of buildings, traditional buildings 13.0%, while duplexes were the least represented at 1.8%. About 52.2% of buildings were serviced by un-tarred roads. Louvre, side-hung and sliding windows were the most common window types at 44.9%, 27.5% and 25.5%, respectively.

**Table 4:** Housing Characteristics

**Characteristics**	**Frequency**	**Percentage**
Building Type		
Traditional courtyard	50	13.0
Rooming	222	57.7
Flat	106	27.5
Duplex	7	1.8
Total	385	100.0
Window Type		
Louvers	173	44.9
Side-hung	106	27.5
Top-hung	8	2.1
Sliding window	98	25.5
Total	385	100.0
Building Accessibility		
No clear path	42	10.9
By tarred road	142	36.9
By un-tarred roads	201	52.2
Total	385	100.0

Kerosene and charcoal were the dominant forms of cooking fuels, used by 92.5% and 66.0% of the population, respectively, followed by firewood (20.5%), while the least used was sawdust *(Table 5).* Moreover, 55.6% of the residents used personal gasoline generators and only one-third (32.7%) of the buildings did not have a generator. Almost 70% of the residents (40.8%; 29.1%) practiced refuse burning and a very small proportion (5.5%) were serviced by garbage collection trucks.

**Table 5:** Domestic Utilities and Practices

**Utilities**	**Frequency**	**Percentage**
Adopted cooking fuel		
Firewood	79	20.5
Sawdust	1	0.3
Charcoal	254	66.0
Kerosene	356	92.5
Gas cooker	132	34.3
Electric cooker	123	31.9
Method of waste disposal		
Dumped in streams	34	8.8
Burnt in a communal chosen open space	112	29.1
Burnt within the premises	157	40.8
Thrown into bushes	61	15.8
Collected by garbage trucks	21	5.5
Total	385	100
Use of generator		
Yes	214	55.6
No	171	44.4
Total	385	100
Generating sets per building		
None	126	32.7
One set	175	45.5
Two sets	59	15.3
Three sets	15	3.9
More than 4 sets	10	2.6
Total	385	100
Use of air conditioners		
Yes	18	4.7
No	367	95.3
Total	385	100

### Spatial variation in PM levels within the study area

To explore the variation of air quality across the residential zones in Ogbomoso, PM_1,_ PM_2.5_ and PM_10_ were merged into a composite index of cumulative particulate matter aggregate (CPMag) used as a surrogate measure of air quality and then analyzed over the 385 sampling points within the 27 precincts across the 3 residential zones using analysis of variance. Table 6 presents the results for the indoor CPMag where the transition zone, had the highest indoor CPMag (83.54 μg/m^3^). Next was the core area with a CPMag of 79.65 μg/m^3^, while the suburban zone had the lowest indoor CPMag level (68.29 μg/m^3^). The observed differences in indoor CPMag across the zones were statistically significant with an alpha level of 0.05 [F_2, 382_ = 25.024; p = 0.0005 (< 0.05)]. Hence, indoor air quality varied significantly within the residential zones. Tukey HSD post-hoc test further explains the variation. While there was no statistically significant difference between the indoor CPMag of the core area and that of the transition zone [p value = 0.304 (> 0.05)], the indoor CPMag of the suburban zone differed significantly from both [p value = 0.005 (> 0.05)]. Since the suburban zone had the lowest indoor CPMag, the zone therefore had significantly better indoor air quality than the other residential zones.

**Table 6:** Indoor PM Aggregate (PM_1_, PM_2.5_ and PM_10_)

**Residential zone**	**No.**	**Mean (log transformation)**	**Mean (μg/m^3^)**	**SD**	**Standard Error**	**95% confidence interval for mean**	

						**Lower bound**	**Upper bound**
Core	73	1.9012	79.65	.08087	.00947	1.8823	1.9200
Transition	187	1.9219	83.54	.10702	.00783	1.9064	1.9373
Suburban	125	1.8407	68.23	.09974	.00892	1.8230	1.8583
Total	385	1.8916	77.91	.10627	.00542	1.8809	1.9022

Variations in the outdoor CPMag are presented in Table 7. The core area had the highest outdoor CPMag level (87.52 μg/m^3^), followed by the transition zone (81.62 μg/m^3^), while the suburban zone had the lowest level (69.68 μg/m^3^). The difference was statistically significant with an alpha level of 0.05 (F_2, 382_ = 7.522; p = 0.01). Hence, outdoor air quality also varied significantly across the residential zones. The Tukey HSD post-hoc test indicated that the outdoor air quality of the suburban zone was significantly better than that of the core area (p = 0.01), while the difference between the mean CPMag scores for suburban and transition zones was not significant [p = 0.06 (>0.05)]. This implies that the suburban zone, although a lower score than the transition area, cannot be said to be significantly better than the transition zone with respect to outdoor CPMag.

**Table 7:** Outdoor PM Aggregate (PM_1_, PM_2.5_ and PM_10_)

**Residential zone**	**No.**	**Mean (log transformation)**	**Mean (μg/m^3^)**	**SD**	**Standard Error**	**95% confidence interval for mean**	

						**Lower bound**	**Upper bound**
Core	73	1.9421	87.52	.18866	.02208	1.8981	1.9861
Transition	187	1.9118	81.62	.20362	.01489	1.8824	1.9412
Suburban	125	1.8431	69.68	.17309	.01548	1.8125	1.8738
Total	385	1.8952	78.56	.19459	.00992	1.8757	1.9147

**Figure 4:**
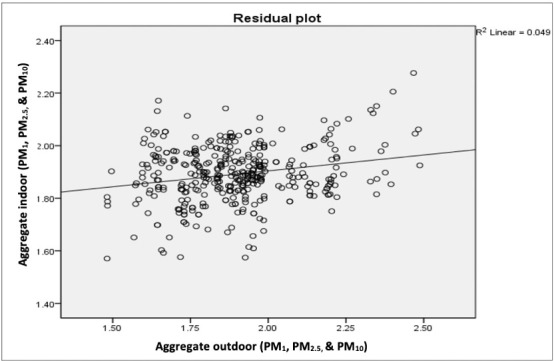
Indoor/outdoor CPMag scatter plot

Correlation analysis was used to explain the relationship between indoor and outdoor air quality. It showed a linear relationship between the indoor and outdoor CPMag with a significant positive correlation of +0.221. This correlation coefficient, which was less than 0.49, was weakly positive.^24^ The positive correlation, although weak, implies that indoor PM level increased without door PM level in the study area. The linear regression model was statistically significant (F_1, 383_ = 19.731; p = 0.0005) and from the regression model *(Figure 4),* the coefficient of determination (R^2^) was 0.049.

## Discussion

As shown in Table 1, the obtained cumulative mean concentrations for indoor and outdoor respirable particles (PM_2.5_) of 41.6±10.1 μg/m^3^ and 46.3±8.0 μg/m^3^ respectively, were lower than the WHO Interim Target 1 limit of 75 μg/m^3^.^25^ It should be noted that the Interim Target 1 used as the baseline is higher than the WHO's air quality guideline levels and was proposed by the WHO Working Group to promote steady progress towards meeting air quality guidelines objectives in developing countries where pollution levels frequently exceed recommended guidelines.^25^ Hence, particle pollution from respirable particles in Ogbomoso was considered to be within the acceptable limit. On the other hand, the cumulative mean concentrations of indoor and outdoor inhalable particles (PM_10_), 175.8±54.3 μg/m and 188.8±122.0 μg/m^3^ respectively, were higher than WHO Interim Target 1 limit of 150 μg/m^3^ for daily averages. These findings are consistent with findings on PM pollution across six selected major cities in Nigeria where the daily PM_10_ mass loads also exceeded the WHO prescribed daily limit but the PM_2.5_values were within the set limit.^19^

Five air quality levels, based on PM_10_ concentration, were established by the Urban Air Quality Management Strategy in Asia, with 0–6 μg/m^3^ categorized as good; 60–120 μg/m^3^ as moderate; 121–350 μg/m^3^ as unhealthy; 351–425 μg/m^3^ as very unhealthy and greater than 425 μg/m^3^ as hazardous.^26^ Using these benchmarks, outdoor air quality in Ogbomoso Township measured via inhalable particles (PM_10_), although not hazardous, can be described as unhealthy. The recorded indoor and outdoor inhalable particles in the study area (175.8 and 188.8 μg/m^3^) were higher than the set limit, although greater concentrations, higher than 300 μg/m^3^, have been recorded in Bangladeshi households as well as in Aba, Port Harcourt, and Lagos–all highly industrialized Nigerian cities.^19,27–29^

### Indoor and outdoor PM levels

**Figure 2:**
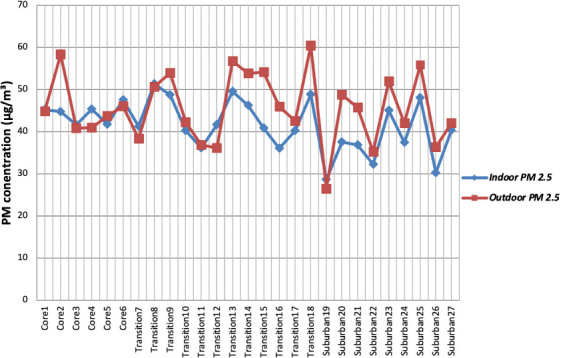
Indoor-outdoor PM_2.5_ levels across residential precincts

**Figure 3:**
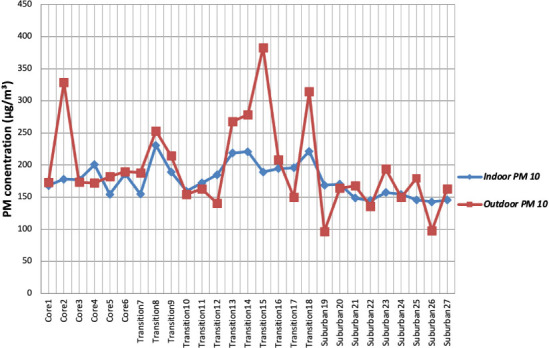
Indoor-outdoor PM_10_ levels across residential precincts

The mean PM_2.5_and PM_10_ levels in Table 1 were plotted across precincts and the trend analyzed in Figures 2 and 3. For both PM_2.5_ and PM_10_, the indoor levels lagged behind, suggesting that outdoor levels of inhalable and respirable particles were higher than the indoor levels. Nonetheless, indoor levels of inhalable particles were higher than the WHO limits and a significant contributor to this was cooking fuel. Most residents use more than one cooking fuel, and cumulatively, kerosene was the dominant cooking fuel used by 92.5% of the population *(Table 3).* Charcoal and firewood were reportedly used in 66.0% and 20.5% of homes, respectively. With biomass fuel predominant in the study area, the resulting emissions were a major source of particulate matter and volatile organic compounds.^30–32^

Although there were points where indoor PM_2.5_ and PM_10_ levels exceeded outdoor levels, the trend showed that air pollution in the ambient outdoor environment was generally greater than pollution within buildings. Outdoor PM sources in the study area, typical of urbanizing traditional towns, include vehicle exhausts along roads (largely service cars, trucks and heavy vehicles), indiscriminate open refuse burning and gasoline generator exhausts from residences or commercial establishments, given the erratic power supply.^10,33^ Most dwellings were connected by an array of distributors and access roads, of which only 36.9% were tarred *(Table 4).* The lack of unpaved roads fosters dust circulation, and hinders efficient road traffic, leading to congestion, vehicle idling and proliferation of exhausts within the environment. Nearly half (40.8%) of residents burn their refuse around their residential premises and one third (29.1%) in communal open dumps *(Table 5)*, making intermittent dumpsite fumes a common occurrence. Moreover, with 67.3% of buildings using gasoline generators, given the inadequate public electricity supply, generator emissions are widespread, causing noise, oxides of carbon, nitrogen, sulfur, and particulate matter.^34^

Figures 2 and 3 also show that PM_2.5_ and PM_10_ concentrations both share a similar pattern from the core area to the suburban area. For both, the levels appear to reach their peak in the transition zone and decrease towards the suburban zone. This is especially true of indoor PM_10_ concentrations which steadily increased in the core area (core 1–4), reached its peak in the transition zone (transition 8) and gradually decreased towards the suburban zone (suburban 25–27). Both the highest levels for indoor PM_2.5_ (51.4 μg/m^3^) and outdoor PM_2.5_ (60.5 μg/m^3^) were recorded in precincts 8 and 18 of the transition zone, respectively. The highest mean indoor PM_10_ (230.8 μg/m^3^) and outdoor PM_10_ (382.7 μg/m^3^) levels were similarly recorded in the precincts of the transition zone, which was also the case for ultrafine particles *(Table 1).*

### Particulate matter variation across residential zones

The spatial pattern of pollution concentrations over a given area depend on the number, type, strength and distribution of various sources, and on the diffusion potential of the atmosphere, as patterns will change somewhat according to prevailing weather conditions. However, the basic underlying pattern of air pollution concentrations over a given area generally remains fairly constant with some areas having generally higher levels than other areas and given a wide enough number of sampling points as in the present study, this pattern can be revealed.^35^ Results from the survey confirmed this notion as it showed that air quality varied within the residential zones in Ogbomoso. As shown in Tables 6 and 7, the lowest indoor CPMag was recorded in the suburban zone (68.29 μg/m^3^) and was statistically different from that of the core (79.65 μg/m^3^) and transition (83.54 μg/m^3^) zones. Similarly, the lowest outdoor CPMag was recorded in the suburban zone (69.68 μg/m^3^) and though not statistically different from the transition zone (81.62 μg/m^3^), was significantly different from that of the core area (87.52 μg/m^3^). Consequently, the transition zone had the worst indoor air quality; the core area, the worst outdoor air quality; while the suburban zone has the best general air quality.

The results can be further explained by residents' domestic utilities and practices which are presented in Table 8, as previously reported.^22^ Burning of refuse is widely practiced by 70% of the residents (*Table 5*), and according to the chi square analysis, this did not vary significantly across the three zones. Hence, other factors like traffic intensity were most likely responsible for the low outdoor air quality in the core area. The core area, as seen in Figure 1, forms the center of an array of distributor and major roads. The chi square test also showed that ownership of gasoline generators varied across residential zones and the transition zone had the highest proportion of generators (45.3%). The majority of these were often situated near dwelling units. Unclean cooking fuels (charcoal and firewood) also exhibited significant variations across the zones with the transition zone again having the highest proportion (45.7% and 43.0%, respectively), perhaps explaining why the zone has the worst indoor air quality. These findings agree with the WHO report that 56% of people in developing countries rely on solid fuels for cooking, the burning of which produces extremely high levels of indoor air pollution and can raise indoor PM_10_ to 3000 μg/m^3^.^6,36^

**Table 8:** Fuel Use and Waste Disposal Across Residential Zones

**Drivers of PM levels**		**Residential zone**			**Chi-square analysis**		

		**Core**	**Transition**	**Suburban**	**X^2^**	**Asymptotic significance**	**Remark**
Waste disposal	In communally chosen spots	14 (12.5%)	60 (54.1%)	37 (33.3%)			
	Around the premises	28 (18.2%)	69 (44.8%)	57 (37.0%)	9.168	0.057	Not significant
	Other disposal methods	31 (25.8%)	58 (48.3%)	31 (25.8%)			
Cooking with kerosene stove	Yes	68 (19.1%)	176 (49.4%)	112 (31.5%)	2.256	0.324	Not significant
	No	47 (15.4%)	153 (50.3%)	106 (34.6%)			
Cooking with charcoal	Yes	65 (25.6%)	116 (45.7%)	73 (28.7%)	21.792	0.0005	Significant
	No	8 (6.1%)	71 (54.2%)	52 (39.7%)			
Cooking with firewood	Yes	26 (32.9%)	34 (43.0%)	19 (24.1%)	12.997	0.020	Significant
	No	55 (21.0%)	135 (51.5%)	72 (27.5%)			
Cooking with electric cooker	Yes	18 (14.6%)	52 (42.3%)	53 (43.1%)	9.540	0.008	Not significant
	No	73 (19.0%)	186 (48.4%)	125 (32.6%)			
Ownership of generator	Yes	35 (16.4%)	97 (45.3%)	82 (38.3%)	7.849	0.020	Significant
	No	38 (22.2%)	90 (52.6%)	43 (25.1%)			

Adopted from Jelili *et al*.^22^

The cumulative PM_2.5_/PM_10_ ratio for the study area was 0.24 (*Table 2*). This ratio is useful for identifying emission sources of particulate matter.^37^ A high PM_2.5_/PM_10_ ratio signifies dominance by fine particles, while a low ratio implies dominance by coarse particles. In other words, the higher the PM_2.5_/PM_10_ ratio, the lesser dominance of coarse particles. This ratio in Ogbomoso was similar to those of Aba (0.18), Lagos (0.29) and Port Harcourt (0.23).^19^ Sampled PM_2.5_/PM_10_ ratios in Nigerian cities revealed that the PM size fraction is dominated by coarse particles, a finding further strengthened by the situation in Ogbomoso.^37^ Using analysis of variance, the difference in the PM_2.5_/PM_10_ ratio across the zones was statistically significant at an alpha level of 0.05 both for indoor (F_2, 382_ = 5.351; p = 0.05) and outdoor (F_2, 382_ = 11.732; p = 0.0005) environments.

The Tukey HSD post-hoc test revealed that the indoor PM_2.5_/PM_10_ ratio of the transition zone was significantly different from that of the core area and suburban zone (p value = 0.036; 0.013), while the outdoor PM_2.5_/PM_10_ ratio of the suburban zone was significantly different from the core and transition zone (p value = 0.0005; 0.002). The transition zone (*Table 2*) had the least indoor PM_2.5_/PM_10_ ratio (0.22), while the suburban zone has the highest outdoor PM_2.5_/PM_10_ ratio (0.28). Hence, it can be deduced that although coarse particles generally dominate the PM size fraction in Ogbomoso, they are significantly higher in the transition zone's indoor environment and lower in the suburban's zone outdoor environment. This conclusion is further corroborated by the trend observed in Figure 3. With the transition zone having a significantly higher proportion of use of gasoline generators and unclean cooking fuels (*Table 8*), such activities were likely responsible for the higher levels of coarse particles.

### Indoor/outdoor PM relationship

The disparity in indoor and outdoor air quality is often explained by the I/O ratio. The hourly average concentrations in the study area were used to calculate the average I/O ratio (*Table 3*), resulting in ratios of 0.84, 0.9 and 0.92 for PM_1_, PM_2.5_ and PM_10_, respectively^.^ These were considerably low, compared to the average I/O ratios of 3.74, 2.88, and 1.71, respectively, for PM_1_, PM_2.5_ and PM_10_ in urban residential sites of Pakistan.^38^ Indoor/outdoor ratios close to unity are considered negligible, and are often due to open windows.^35^ Three mechanisms influence I/O airflow, including mechanical ventilation, natural ventilation and infiltration.^9^ Natural ventilation permeates buildings primary via windows and through doors, cracks and other openings.^39^ Windows particularly enable pollutant penetration into the indoor space, as residents open them to allow as much air as possible into buildings, given the prevailing hot climate typical of the tropics.

It is noteworthy that 53% of the population used sliding windows and side-hung (25.5%; 27.5%), window types that allow for unobstructed inflow of air *(Table 4).* These windows are more rigid and less effective for controlling ventilation, unlike louvers that have mechanisms for managing air flow and are slightly more effective *(Table 9).* The dominance of the rooming building type (57.7%) exacerbates the situation. These are dwellings with a long narrow passage separating rows of directly opposite rooms with single widows. This situation, coupled with the poor public power supply, relative absence of mechanical ventilation and in filtration from building cracks or leaks, allows outdoor air pollutants to easily penetrate the indoor environment, which could narrow the I/O ratio. Enquiries into the influence of ventilation systems on indoor particle concentration shows that I/O ratios range from 0.25–0.60 for particles larger than 1 μm (PM_2.5_ and PM_10_) in apartments with natural ventilation.^40^ This is not far off from the 0.82–0.98 range (*Table 3*) observed in the study area.

**Table 9:** Window Properties

**Type**	**Diagram**	**Basic features**	**Percentage in study area**
Louvers	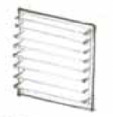	Wide range of opening Blades direct air flow into space Very effective for ventilation	44.9%
Side-hung	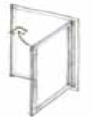	Fixed range of opening (up to 90°) Effective for ventilation 100% of aperture unobstructed	27.5%
Top-hung	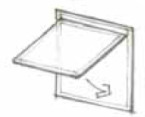	Fixed range of opening (up to 90°) Less effective for ventilation	2.1%
Sliding window	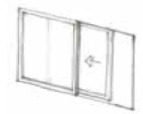	Limited range of opening (about 50% of aperture size) Less effective for ventilation	25.5%

Adapted from Building Planning and Massing.^41^

**Figure 5:**
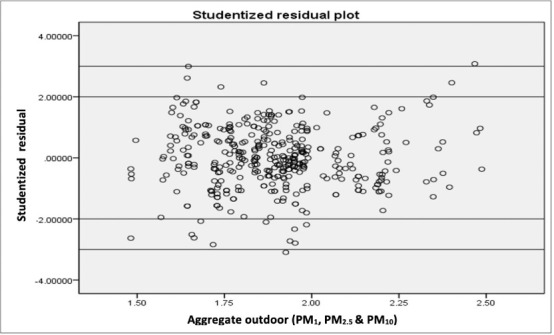
Indoor/outdoor CPMag studentized residual plot

There was a weak but significant positive linear relationship between indoor and outdoor CPMag as revealed in the correlation analysis. Indoor PM level increased with outdoor PM level in the study area. The resulting coefficient of determination (R^2^) was, however, far from 1 *(Figure 4),* so the resulting regression equation was not useful for making predictions. All the underlying assumptions for regression analysis were met as the studentized residual plot shows a random scatter of the points i.e. independence with a constant spread, thus, constant variance and no values beyond the ±3 SD reference lines (no significant outliers), as seen in Figures 4 and 5.

Consequently, the low R^2^ in the results implies that outdoor PM aggregate does not explain much of the variability in the indoor PM aggregate. Only 4.9% (≈ 5%) of the total variation in indoor PM aggregate was associated with outdoor PM aggregate. The low R^2^ (5%) in Ogbomoso was unlike the findings for the German city of Erfurt where the R^2^ was 86%, showing very strong correlations for indoor and outdoor respirable particles (PM_2.5_) with outdoor PM_2.5_ levels explaining most (80%) of the indoor variation of PM_2.5_.^43^ However, the aforementioned study was carried out under more controlled conditions as no indoor sources of particles were present in the rooms and no human activities occurred during the measurement periods in contrast to the setting in Ogbomoso. With only 5% of the total variation in indoor PM levels associated with those of outdoor levels, the remaining 95% can only be accounted for by indoor or within-building factors such as building types and materials, and more importantly indoor utilities and cooking practices. It is thus reasonable, given the low R^2^ value, to suggest that although outdoor PM levels significantly affect indoor levels, indoor activities (domestic heating and cooking) were the major sources of emissions impairing indoor air quality in the study area.

## Conclusions

Air quality is an important factor in safeguarding human health, thus the present study analyzed airborne particulate matter facets (ultrafine, inhalable and respirable), zone-specific facets (core, suburban and transition zone) and indoor-outdoor facets. Air quality was relatively poor in Ogbomoso, especially with regard to inhalable particles. Only the suburban part of the town had good air quality. While the current situation was not as hazardous as some industrialized cities within and outside the country, PM should be kept from reaching hazardous levels.

Given the situation in the study area, appropriate measures are needed to improve air quality, especially indoor air quality. Considering the observed intra-urban variation of PM levels, efforts should be prioritized in the transition zone. While outdoor air pollution affects indoor air quality owing to infiltration and ventilation, indoor activities appear to contribute more to indoor air pollution than outdoor sources. Hence, good indoor air quality is best achieved by dealing with domestic utilities and practices within traditional urbanizing settlements. A good place to begin such efforts would be a shift from the prevalent use of biomass cooking fuels (firewood, charcoal, sawdust) and personal generator sets to cleaner energy sources. This can be achieved by revitalizing hydroelectricity and improving access to alternatives like liquefied petroleum gas and solar power.

Effective waste management is also imperative. Sanitary landfills are a safer and healthier means of refuse disposal and should be adopted, while open dumps should be discouraged, and stringent measures enforced where needed. The transportation system within the city could also be improved by rehabilitating poorly tarred and un-tarred roads to reduce vehicle idling and dust propagation. Most importantly, the inter-city commute of commercial heavy trucks through the town should be rerouted via the outskirts by construction of alternate express ways, like ring roads, to prevent unnecessary traffic congestion within the town. These steps to be taken by local and state governments can mitigate pollutant emissions from domestic and vehicular combustion processes in the study area. It is hoped that the insights provided in the present study will help foster a better understanding and promotion of cleaner ambient air quality in urban areas.
