# Supplementary material for: Comparative Analysis of Indoor and Outdoor Particulate Matter Concentrations and Air Quality in Ogbomoso, Nigeria
Source: J Health Pollut. 2020 Nov 19;10(28):201205. doi: 10.5696/2156-9614-10.28.201205 (PMC7731495; doi:10.5696/2156-9614-10.28.201205)
Supplement: Supplementary file 1 [file Alabi_Supplemental.docx]

**Supplemental Material**

**Ladoke Akintola University of Technology, Ogbomoso, Oyo State**

**Faculty of Environmental Sciences,**

**Department of Urban and Regional Planning**

**Intra-Urban Pattern of Air Quality in Ogbomoso**

Dear Respondent,

This questionnaire is designed to collect information on air quality within Ogbomoso. It is strictly for academic purposes and your willingness to provide truthful and accurate information for this research is highly valued.

Thank you for your cooperation.

Abimbola T. Alabi

**Part A (For the respondent)**

**Socioeconomic characteristics**

1. Dwelling location? _________________________________________________
2. Gender? (i) Male (ii) Female
3. Age of respondent? (i) 18-39 (ii) 40-59 (iii) 60 and above
4. Household size? _____________________________
5. Your level of education? (i) No formal education (ii) Primary education (iii) Secondary education (iv) Post-secondary education (v) Tertiary education
6. Major Occupation? (i) Artisan (ii) Farming (iii) Trading (iv) Teaching (v) Civil service (vi) Other professional services (vi) Unemployed
7. Average income per month (i) Below #18,000(ii) #18,000-#50,000 (iii) #51,000-#100,000 (iv) #100,000-#200,000 (v) Above #200,000

**Domestic practices**

1. Tick the any of the following cooking energy sources used in your home

| Cooking Energy | Yes | No |
| --- | --- | --- |
| Firewood |  |  |
| Charcoal |  |  |
| Kerosene stove |  |  |
| Gas cooker |  |  |
| Electric cooker |  |  |
| Sawdust |  |  |

1. Where do you cook your food? (i) In the room (i) In the passage (ii) Behind the building (iii) In the kitchen
2. How are domestic wastes disposed of? (i) Tipped into stream/drains (ii) Burnt in a communally chosen place (iii) Burnt within the premises (iv) Thrown into bushes (v) Collected by government agencies
3. Do you use an air conditioner at home (i) Yes (ii) No
4. Floor covering? (i) Rug (ii) Carpet (iii) Tiles (iv) Terrazzo (v) Concrete {None}
5. Ceiling type? (i) None (ii) Unpainted asbestos (iii) Painted asbestos (iv) Planks (v) P.O.P
6. Do you own a generator (i) Yes (ii) No
7. How many generators are used in your building? (i) None (ii) 1 (iii) 2 (iv) 3 (v) 4 (vi) Above 4
8. How long do you use your generator daily? (i) Less than 2 hrs (ii) 2-6 hrs (iii) 6-10 hrs (iv) over 10 hrs

**General**

1. Is there any business enterprise using large generators close by? (i) Yes (ii) No
2. How often do you perceive tobacco smoke in your area? (i) Not at all (ii) Rarely (iii) Daily
3. How often do you use the following domestic utilities?

| Variable | Not at all | Not Frequently | Moderately | Frequently | Very Frequently |
| --- | --- | --- | --- | --- | --- |
| Insecticides/Pesticides |  |  |  |  |  |
| Generator |  |  |  |  |  |
| Perfumes/Deodorant |  |  |  |  |  |
| Air fresheners |  |  |  |  |  |
| Mosquito coil |  |  |  |  |  |
| Coal iron |  |  |  |  |  |
| Incense |  |  |  |  |  |

1. How often do you adopt the following light and cooking energy sources?

| Variable | Not at all | Not frequently | Moderately | Frequently | Very frequently |
| --- | --- | --- | --- | --- | --- |
| Sawdust |  |  |  |  |  |
| Firewood |  |  |  |  |  |
| Charcoal |  |  |  |  |  |
| Kerosene stove |  |  |  |  |  |
| Electric cooker |  |  |  |  |  |
| Gas cooker |  |  |  |  |  |
| Lantern |  |  |  |  |  |
| Candle |  |  |  |  |  |

**Part B (For the Researcher)**

**Housing/environmental characteristics**

1. Building type (i) Impluvium (ii) Rooming (iii) Flat (iv) Duplex
2. Wall type (i) Mud (ii) Cement block (iii) Mud bricks
3. Ambient floor material (i) Earth (ii) Plank (iii) Concrete (iv) Tiles
4. Window type (i) Wood (ii) Iron (iii) Louvre (iv) Top hung (v) Sliding (vi) Side hung
5. Window size (i) 1.2 m by 0.8 m (ii) 1.2 m by 1.2 m (iii) 1.2 m by 1.6 m (iv) 1.2 m by 1.8 m
6. Roof structure (i) Asbestos (ii) Iron/aluminium roofing sheets (iii) Decking
7. Roof condition (i) Leaking (ii) Sagging (iii) No defect
8. Use of building (i) Only residential (ii) Residential & commercial (iii) Residential & industrial (iv) Residential & commercial & industrial
9. Specific adjoining mixed use _______________________________________
10. Number of trees around the compound? _________________
11. Observed open dumps along the street? _________________
12. Building accessibility (i) No clear path (ii) By tarred road (iii) By un-tarred road
13. Pollutant concentration within the house
14. General condition of the environment (i) Very clean/tidy (ii) Fairly clean/tidy (iii) Poor/filthy/stinking(iv) Very poor/filthy/stinking
15. Indoor pollutant levels

| **AQ Parameters** | **Readings** | **Mean** |
| --- | --- | --- |
| PM_1_ |  |  |
| PM_2.5_ |  |  |
| PM_4_ |  |  |
| PM_7_ |  |  |
| PM_10_ |  |  |
| TSP |  |  |

1. Outdoor pollutant levels

| **AQ Parameters** | **Readings** | **Mean** |
| --- | --- | --- |
| PM_1_ |  |  |
| PM_2.5_ |  |  |
| PM_4_ |  |  |
| PM_7_ |  |  |
| PM_10_ |  |  |
| TSP |  |  |
